# Associations of prior wasting malnutrition with later indicators of glucose tolerance across 4 countries in Africa and Asia: The Severe Acute Malnutrition - the Role of the Pancreas (SAMPA) study

**DOI:** 10.1016/j.ajcnut.2025.07.032

**Published:** 2025-08-05

**Authors:** Dixi Paglinawan Modoc, Sana Ahmed, Molly Chisenga, Sharon E Cox, Riddhi Dasgupta, Paulita Duazo, Daniel Faurholt-Jepsen, Lackson Kasonka, Paul Kelly, Ruth Keogh, Belinda Kweka, Rikke Krogh-Madsen, Nanette Lee, Evangelista Malindisa, Dorothea Nitsch, Patrick Ngoya, George PrayGod, James AM Shaw, Juan Antonio Solon, Mizinga Jacqueline Tembo, Geeta Trilok-Kumar, Suzanne Filteau

**Affiliations:** 1 Nutrition Center of the Philippines, Manila, Philippines; 2 Institute of Home Economics, University of Delhi, New Delhi, India; 3 University Teaching Hospital, Lusaka, Zambia; 4 Faculty of Epidemiology and Population Health, London School of Hygiene and Tropical Medicine, London, United Kingdom; 5 School of Tropical Medicine and Global Health, Nagasaki University, Nagasaki, Japan; 6 Translational and Clinical Research Institute, Newcastle University, Newcastle upon Tyne; 7 University of San Carlos-Office of Population Studies Foundation, Inc, University of San Carlos, Cebu, Philippines; 8 Department of Infectious Diseases, Rigshospitalet, Denmark and Department of Clinical Medicine, University of Copenhagen, Denmark; 9 Blizard Institute, Barts and The London School of Medicine and Dentistry, Queen Mary University of London, London, United Kingdom; 10 Tropical Gastroenterology and Nutrition group, University of Zambia School of Medicine, Lusaka, Zambia; 11 Mwanza Research Centre, National Institute for Medical Research, Mwanza, Tanzania; 12 Centre for Physical Activity Research, Rigshospitalet, University of Copenhagen, Copenhagen, Denmark; 13 Department of Physiology, Catholic University of Health and Allied Sciences, Mwanza Tanzania; 14 Department of Radiology, Bugando Medical Centre, Catholic University of Health and Allied Sciences, Mwanza, Tanzania; 15 Trivedi School of Biosciences, Ashoka University, Haryana, India; 16 Newcastle Centre for Diabetes Care, Newcastle upon Tyne Hospitals NHS Foundation Trust, Newcastle upon Tyne; 17 Department of Infectious Diseases, Copenhagen University Hospital, Copenhagen, Denmark

**Keywords:** diabetes, wasting, cohort, oral glucose tolerance test, hemoglobin A1c

## Abstract

**Background:**

Prenatal or infant wasting malnutrition followed by later overweight is associated with increased risk of chronic diseases, including type 2 diabetes.

**Objectives:**

In a pooled analysis of 6 longitudinal cohorts, we investigated associations between prior malnutrition (PM) early in life or in adulthood and subsequent glycemic status.

**Methods:**

We identified cohorts in Tanzania, Zambia, India, and the Philippines in whom low birth weight or wasting malnutrition in childhood or as adults following human immunodeficiency virus or tuberculosis infection had been measured. Anthropometry, body composition, and glycemic status, determined by hemoglobin A1c (HbA1c), and glucose at 120 min in an oral glucose tolerance test (glucose120), were assessed 3–38 y after PM and in non-PM (NPM) controls. HbA1c and glucose120 were compared between PM and NPM participants by linear regression, controlling for age, sex, and socioeconomic status and, in pooled analyses, for cohort also.

**Results:**

In the full cohort of 2251 participants, there was no overall association between PM and diabetes risk. Child participants aged ∼12 y who were hospitalized with PM when <2 y had higher glucose120 compared to NPM (difference 0.50 mmol/L; 95% CI: 0.10, 0.91 mmol/L). In pooled analyses across adult cohorts controlling for cohort, age, sex, and socioeconomic status, PM participants, compared to NPM, may have higher glucose120 (difference 0.33 mmol/L; 95% CI: –0.27, 0.92 mmol/L) if still underweight, and higher HbA1c (difference 0.41%; 95% CI: –0.07%, 0.89%) and glucose120 (difference 0.70 mmol/L; 95% CI: –0.25, 1.66 mmol/L) if currently obese.

**Conclusions:**

Childhood PM is associated with greater adult dysglycemia, whereas adulthood PM may have heterogeneous outcomes dependent on subsequent presence/absence of weight gain. Clinicians and public health managers should be aware of the long-term risk and intervene to promote some weight gain but prevent excess weight gain in people who were previously malnourished.

## Introduction

With the nutrition transition, the prevalence of obesity and overweight is increasing even in low- and middle-income countries (LMICs) where wasting malnutrition, both among young children and adults, including those with severe infections such as HIV and tuberculosis, remains prevalent [1]. Being overweight alone can increase the risk of noncommunicable diseases (NCDs), including non-type 1 diabetes [2]. In addition, there is evidence that wasting malnutrition may, in both the short and long term, alter glucose metabolism and increase diabetes risk [3].

The capacity-load model hypothesizes that, when malnutrition earlier in life is followed by obesity in later life, the risk of NCDs may increase [4]. Most evidence for the capacity-load model is from prenatal malnutrition, usually indicated by low birth weight [4]. However, there is evidence that childhood or later wasting malnutrition combined with subsequent weight gain may increase the risk of some NCDs. A recent systematic review [3] reported that wasting malnutrition experienced during childhood or adulthood may be associated with impaired pancreas function later in life. Most clinical studies in this review were conducted decades ago and were often not conducted or reported to the standard now expected. A study of adults who survived the mid-twentieth-century Chinese famine found that the risk of diabetes was highest in those with currently higher BMI (in kg/m^2^) or eating a more Western, rather than a traditional Chinese, diet [5]. Our research in Tanzania has demonstrated an association of prior wasting malnutrition with reduced insulin production during an oral glucose tolerance test (OGTT) in adult males, most of whom had normal (18.5–25.0) or low (<18.5) BMI at the time of the OGTT [6].

Most wasting malnutrition at any age is due to a combination of factors, with inadequate diet and severe or repeated infections being the interacting proximal causes. This is best established for childhood wasting malnutrition [7], but severe infections in adulthood can also cause malnutrition. Anorexia nervosa is an exception since infection is not a key component of the malnutrition, but in the present context, it is interesting that patients with anorexia nervosa, after nutritional rehabilitation, had poor glucose tolerance compared with BMI-matched controls [8]. The similarity between this article [8] and others on malnutrition associated with infections, or poverty in the systematic review [3] suggests that malnutrition, whatever its cause, may impair pancreatic endocrine function. This mechanism may contribute to the phenotype of diabetes in some LMICs where, unlike the common situation in high-income countries, diabetes frequently develops at an earlier age and may do so at a normal or low BMI [9].

Severe Acute Malnutrition - the Role of the Pancreas (SAMPA) was designed to study several aspects of pancreas structure and function among people previously malnourished (PM), irrespective of cause, or not previously malnourished (NPM). The underlying hypothesis of the present work was that PM across all stages of life, from in utero to adulthood, is associated with increased risk of diabetes. We investigated this hypothesis using established cohorts in Zambia, Tanzania, India, and the Philippines for whom we had previously measured nutritional status with confirmed PM, and for whom we had appropriate NPM comparators in each setting. A cohort study permitted us to improve on prior cross-sectional and famine follow-up studies since we had access to research-quality anthropometric data both during malnutrition and many years later. We also tested whether later overweight (3–38 y later) accentuates the effects on glucose metabolism resulting from PM.

## Methods

### Study populations

The SAMPA study is registered at clinicaltrials.gov as NCT05361148 and has a published protocol describing in detail the study design, methods, cohorts, and participant flow charts since recruitment [10]. The study was a follow-up of 6 diverse cohorts from India (Delhi Infant Vitamin D Supplementation Study [DIVIDS]) initial recruitment at birth 2007–2010 [11], Zambia (Severe Acute Malnutrition [SAM]), hospitalized for malnutrition at <2 y of age between 2010 and 2014 and recruited with NPM controls 2018–2020 [12], and (Nutritional Support for African Adults Starting Antiretroviral Therapy [NUSTART]), initial recruitment as adults 2011–2013 [13]; Tanzania (Chronic Infections, Comorbidities and Diabetes in Africa [CICADA]), initial recruitment as adults 2006–2017 [14]; and Philippines (Starting Anti-TB Treatment [St-ATT]), initial recruitment as adults 2018-2020 [15], and (Cebu Longitudinal Health and Nutrition Survey [CLHNS]), initial recruitment at birth 1983–1984 [16]. Participants come from LMICs at varying stages of the nutrition transition but with persistent infectious disease burdens. Cohorts include those with (PM) or without (NPM) documented prior exposure to wasting malnutrition.

Participant contact details from their most recent follow-ups were used to call and recruit cohort members for further follow-up assessment. In cases where valid contact information was not available, home visits were conducted to facilitate recruitment. For all cohorts except DIVIDS and CLHNS, we recruited all available and consenting participants. In DIVIDS, logistical issues interfered with recruitment. The subsample for CLHNS was recruited as detailed below. SAMPA participants came for study visits between October 2021 and August 2023. PM was defined slightly differently for each cohort and was assessed at different times before SAMPA data collection [10].

#### Cohorts describing the impact of low birth weight or PM in childhood

DIVIDS participants were born low birth weight (<2.5 kg) at term and were originally recruited at birth, 2007–2011; NPM controls of similar age were recruited later. PM participants in SAM, born 2009–2013, were hospitalized for SAM at <2 y of age; NPM controls had no record of prior malnutrition and were born 2007–2013. CLHNS participants are from a Filipino birth cohort recruited in 1983–1984 and followed up regularly since. PM in CLHNS was defined as weight-for-length *z*-score (WHZ) <–3 before 2 y of age. All participants experiencing PM when under 2 y were eligible, plus NPM controls, who always had a WHZ >–2 until 2 y of age, randomly selected from the main databases.

#### Cohorts who experienced adult PM

In CICADA, NUSTART, and St-ATT cohorts, PM was assessed in adulthood, was due mostly to tuberculosis and/or HIV infection, and was defined as BMI <18.5 [17]. The CICADA cohort [14] comprised 3 groups: *1*) patients with tuberculosis and controls without tuberculosis, both with known HIV status, originally recruited 2006–2009; *2*) participants in the NUSTART trial conducted in both Mwanza, Tanzania, Lusaka, and Zambia with patients with HIV infection recruited 2011–2013; *3*) HIV-infected and uninfected participants recruited 2016–2017. The NUSTART cohort in SAMPA comprised the Zambian participants in the same NUSTART trial plus more recently recruited NPM controls, with or without HIV infection. The St-ATT cohort was originally recruited in 2018–2020 for a study of diabetes and tuberculosis.

HIV was routinely tested for only in the African cohorts; all participants were stable on antiretroviral therapy at the time of diabetes marker assessment. Methods for assessing tuberculosis disease at the time of initial cohort recruitment varied across cohorts: it was formally diagnosed only in St-ATT, where all participants were infected so there were no uninfected controls, and in some CICADA participants [18]; in NUSTART, tuberculosis was not tested by the research team, but participants who reported being on anti-tuberculosis treatment at recruitment were considered to have tuberculosis [19]; other cohorts were not tested for tuberculosis. Since tuberculosis is a treatable disease, participants no longer had active disease at the time of diabetes assessment.

### Diabetes tests

We used 2 markers of glucose metabolism: hemoglobin A1c (HbA1c) and glucose at 120 min (glucose120, mmol/L) in a standard OGTT. Glucose was measured in a 2-h 3-sample OGTT in children (0 min, 60 min, and 120 min) and a 2-h 7-sample OGTT in adults (0 min, 15 min, 30 min, 45 min, 60 min, 90 min, and 120 min). Participants arrived fasting and were given 75 g of glucose monohydrate (in children, ≤75 g at 1.75 g/kg body weight) in 250 mL water within 5 min of drawing the fasting blood sample. Glucose concentration during the OGTT was measured using point-of-care devices (Hemocue) in all cohorts except DIVIDS, where children were reluctant to have fingerprick blood taken, so glucose was measured in venous blood in a commercial laboratory (Dr Lal PathLabs, Delhi). The Hemocue 201RT device has a published detection range of 0.55–27.8 mmol/L and returns an error message outside this range. Glucose concentrations above the detection limit were set at the upper detection limit, and values below the detection limit were assigned a value equal to half of this lower limit. For descriptive purposes, we categorized diabetes using WHO criteria where glucose120 <7.8 mmol/L indicated normal glucose tolerance, ≥7.8–<11.1 mmol/L indicated prediabetes, and ≥11.1 mmol/L indicated diabetes [20]. Those with an OGTT indicative of diabetes were called for a repeat OGTT to determine whether they needed a clinical care referral.

HbA1c was estimated in fasting blood samples using point-of-care devices (SAM, NUSTART, and CICADA by Hemocue; DIVIDS by Tosoh-G8, Tosoh Corporation; St-ATT and CLHNS Tri-Stat, Trinity Biotech). The published ranges for Tri-Stat devices are 4–14%. Similar to glucose, if a machine returned an error message indicating values above or below the limit of detection, values were set to 2–14%, respectively. HbA1c was categorized as <5.7% for normal, ≥5.7% and <6.5% for prediabetes, and ≥6.5% for diabetes [21].

### Anthropometry

Height, weight, and waist circumference (WC) were measured in duplicate by standard methods [22] and means used in analyses. BMI was calculated as weight divided by height squared. For adults, BMI was categorized as underweight, <18.5; normal weight, ≥18.5 and <25; overweight, ≥25 and <30; and obese, ≥30. We used the same cut-offs for all adult cohorts, although there is some evidence that Asians may be overweight at lower BMI levels. For children, BMI *z*-scores were calculated using Stata zanthro with the WHO growth reference and categorized as underweight, <–1 SD (combining grades 1, 2, and 3 thinness); normal, ≥–1 and <1; overweight, ≥1 and <2; and obese, ≥2.

### Body composition

Body composition was assessed for all cohorts except St-ATT (which lacked the equipment) using bioelectrical impedance (CICADA and NUSTART: Tanita BC-418 segmental analyzer; CLHNS: Tanita MC780 segmental analyzer). Fat mass and fat-free mass (kilogram) were divided by height (square meters) to calculate fat mass index (FMI) and fat-free mass index (FFMI).

### Data management and statistical analyses

Data were collected in REDCap and checked for missing data and outlying values. If found, data queries were sent to cohort managers for verification. Missing codes were added, and outlying values were retained if determined to be accurate. Further cleaning and analyses were done using both Stata 18 (StataCorp) and R (R Core Team 2024; https://www.R-project.org/). Glucose120 data were missing in 5 participants because of feeling ill or absconding partway through the OGTT. HbA1c was missing in 24 participants, mainly due to equipment problems or lack of cuvettes at the site. Participants were included in analyses for which they had data.

We compared participant characteristics of those recruited and the original recruited cohort participants. We analyzed HbA1c and glucose120 primarily as continuous variables. We calculated total area under the glucose concentration curve (AUC) during the OGTT and compared this, HbA1c, and glucose120 between PM and NPM participants by linear regression with robust SE.

Analyses were by linear regression, conducted separately by cohort and controlled for age, sex, and socioeconomic status (SES) tercile. The SES index was constructed using principal component analysis based on household ownership of durable goods and assets. For adult cohorts with available data, we also adjusted for infection status (HIV or tuberculosis) to investigate whether these confound associations. Additional confounders were not considered, largely because of data not being available or being inconsistent across cohorts; however, control for cohort in combined analyses effectively controlled for differences in, for example, time since PM, underlying cause of PM. Residual analyses were conducted to assess linear regression model assumptions. We did not see any severe violations of assumptions as depicted in residual plots. A few models showed deviation from the normality assumption, but we do not consider this a serious problem since we have large enough sample sizes per cohort. A few models show slight deviations from the homoskedasticity assumption, prompting the use of robust SE. We assessed the leverage of each observation; there were a few points at >3 times the leverage, all of which were validated and thus retained in the model. Lack of multicollinearity was confirmed by very low values of the variance inflation factor. We used 2-sided significance testing. We also used standard HbA1c-based or glucose120-based diabetes categories [20,23] for descriptive purposes and to estimate the relative risk (RR) of diabetes or prediabetes among PM compared with NPM participants. We used the generalized linear models in Stata 18 with a binomial family and log link when estimating the RR of elevated HbA1c (≥5.7%) or glucose120 (≥7.8 mmol/L) associated with PM. For the nonconverging log-binomial model, we used the robust Poisson model to estimate the RR.

As a secondary analysis of the continuous variables, glucose120 and HbA1c, we conducted a meta-analysis using the metaan command in Stata. This enabled us to include the HIV status of the African cohort in the models in addition to age, sex, and SES.

Because prior research indicates PM may interact with sex in its associations with later outcomes [3,6], we first checked *P*-values for interactions between sex and PM for associations with HbA1c or glucose120. We also wished to determine whether any association between PM and HbA1c or glucose120 differed according to current nutritional markers - BMI, WC, and FMI – which may be on the causal pathway between PM and later diabetes. For these analyses, we used the adult cohorts combined in order to increase statistical power, but excluded the child cohorts since there were very few cases of abnormal glucose markers among the children. BMI was categorized for both males and females according to standard categories [17]. FMI and WC normally differ between sexes, so sex-specific terciles were generated using the cohorts together and then combined. Since we did not have data on HIV or prior tuberculosis disease for all cohorts, these variables were not included in pooled analyses across cohorts; however, the cohort itself is a proxy for the underlying cause of PM as well as the duration of follow-up. We then conducted linear regressions, using robust SE, including interactions between PM and each marker of current nutritional status, controlling for cohort, age, sex, and SES. Adjusted differences between PM and NPM at each level of current nutritional status are presented as forest plots.

### Sample size and statistical power

Sample size was limited to those available from the ongoing cohorts. In published studies, the absolute difference in prevalence of hyperglycemia in those with PM compared with those without was 3–9% [5,24,25]. In our original proposal, we based power calculation on the cohort, CICADA, for which we had recent relevant data. Assuming a prevalence of 7% in those without malnutrition, the CICADA cohort individually had well above 80% power to detect a difference in prevalence of 7.5% in those with PM, as did some other cohorts, e.g., St-ATT. Once outcome data were obtained, we realized that there was great heterogeneity across cohorts, so that CICADA prior data did not apply to all.

### Ethics

Ethical approval was obtained from the ethics committees of the London School of Hygiene and Tropical Medicine, the United Kingdom (22792-2); National Institute for Medical Research, Tanzania (NIMR/HQ/R.8a/Vol.IX/3686); University of Zambia, Zambia (1686-2021); Institute of Home Economics, University of Delhi, India [IHE/2021-22/Admin/Ethics.com/684(A)]; and St. Frances Cabrini Medical Center - Asian Eye Institute, Philippines (2021-012). Adult participants gave written informed consent. Children gave assent, and their parents gave written informed consent. Participants found to have diabetes or other medical conditions were provided with referrals to local health care facilities.

### Results

The study included 2251 participants: 430 children and 1821 adults (Supplemental Figure 1); losses from original cohort recruitment up to the previous follow-up are published [10]. Reasons for participant loss since the original studies were loss of contact and, for some cohorts, high mortality. Logistical issues interfered with DIVIDS recruitment to SAMPA, and fears of COVID-19 made some potential participants, mainly from St-ATT, reluctant to attend the study clinic. Compared with those not included, participants included in SAMPA from the DIVIDS and NUSTART cohorts were of higher education or SES, whereas the opposite was the case for St-ATT and CLHNS. In CICADA (those recruited at the CICADA baseline) and NUSTART, those included had higher original BMI (Supplemental Table 1). The characteristics showing differences were already planned as covariates or for stratified analyses.

Table 1 summarizes participant characteristics, divided by cohort and whether or not they were PM or NPM. Proportions of males and females were similar in the child cohorts, but the Zambian and Tanzanian adult cohorts included more females than males, and the Filipino cohorts had more males than females. The age range for the currently adult cohorts was similar, around 40–50 y, and the child cohorts were also of similar age: 12–13 y. The interval between having had PM and being followed up differed between cohorts; CLHNS was the cohort with the longest follow-up since PM at age <2 y, and St-ATT had the shortest follow-up since tuberculosis infection. CLHNS participants in general had the highest education level as well as the highest current BMI, and prevalence of overweight and obesity. For all cohorts except DIVIDS, the PM participants had lower current BMI than the NPM. As expected, females had higher FMI than males. There appeared to be larger absolute differences between PM and NPM in FMI for females than for males, but the differences in FFMI were similar for males and females. Although healthy adult males are expected to have higher WC than healthy females, WC was similar between sexes in the Filipino cohorts, and higher in females in the African cohorts.

**TABLE 1:** Description of study populations (column percentages are shown unless otherwise indicated)^1^, ^2^, ^3^.

	DIVIDS, India		SAM, Zambia		CICADA, Tanzania		NUSTART, Zambia		St-ATT, Philippines		CLHNS, Philippines	
Age at PM diagnosis	birth		<2 y		Adult		Adult		Adult		<2 y	
Years since PM diagnosis	∼12		∼10		5–18		8–11		∼3		36–38	
Past nutritional												
Status	NPM	PM	NPM	PM	NPM	PM	NPM	PM	NPM	PM	NPM	PM
*N*	58	264	47	61	637	337	87	201	136	115	201	107
Proportion	28	134	26	32	392	175	75	111	41	34	73	38
female, # (%)	(48)	(51)	(55)	(52)	(62)	(52)	(86)	(55)	(30)	(30)	(36)	(36)
Age, years,	11.9	13.9	13.2	12.2	47.4	48.9	39.9	45.3	51.4	48.1	39.1	38.9
mean (SD)	(1.4)	(0.8)	(2.0)	(1.0)	(11.4)	(10.4)	(12.6)	(7.5)	(14.8)	(16.5)	(0.4)	(0.5)
Known HIV-	-	-	0	2	331	270	39	201	-	-	-	-
infected, # (%)				(3)	(54)	(82)	(45)	(100)				
SES terciles, # (%)												
Low	12	90	14	22	180	145	34	62	45	43	62	41
	(21)	(36)	(30)	(36)	(28)	(43)	(39)	(31)	(33)	(37)	(31)	(38)
Medium	26	78	15	21	221	106	23	73	48	37	69	34
	(46)	(31)	(32)	(34)	(35)	(31)	(26)	(36)	(35)	(32)	(43)	(32)
High	18	82	18	18	236	86	30	66	43	35	70	32
	(32)	(33)	(38)	(30)	(37)	(26)	(35)	(33)	(32)	(30)	(35)	(30)
Current BMI^3^, kg/m^2^, mean (SD)												
Males	16.5	16.8	16.8	15.7	23.0	19.2	22.6	19.1	22.3	18.0	27.5	24.7
	(4.0)	(3.2)	(2.2)	(1.3)	(3.5)	(2.4)	(4.0)	(2.7)	(2.9)	(2.2)	(4.3)	(4.0)
Females	17.2	17.7	18.5	17.7	25.9	20.7	26.6	21.4	23.1	18.3	28.7	25.0
	(3.2)	(3.5)	(3.5)	(3.0)	(5.6)	(3.4)	(6.4)	(4.1)	(3.6)	(2.8)	(5.0)	(4.9)
BMI categories, # (%)												
Underweight	26	128	15	22	21	121	0	73	13	68	0	4
	(46)	(49)	(32)	(36)	(3)	(36)		(37)	(10)	(59)		(4)
Normal	24	114	29	35	366	194	46	106	99	47	55	58
	(43)	(44)	(62)	(57)	(58)	(58)	(53)	(53)	(73)	(41)	(27)	(54)
Overweight	5	14	3	4	160	19	19	15	23	0	92	32
	(9)	(5)	(6)	(7)	(25)	(6)	(22)	(7)	(17)		(46)	(30)
Obese	1	3	0	0	90	3	22	6	1	0	54	13
	(2)	(1)			(14)	(1)	(25)	(3)	(1)		(27)	(12)
FMI, kg/m^2^, median (IQR)												
Males	1.2	1.3	2.5	2.2	3.6	2.2	3.9	2.5	-	-	5.4	4.6
	(0.6, 2.0)	(0.4, 3.0)	(2.4, 3.1)	(2.1, 2.7)	(2.2, 5.7)	(1.4, 3.2)	(2.5, 5.5)	(1.8, 3.4)			(3.6, 7.5)	(2.8, 5.6)
Females	3.4	3.6	4.1	3.8	8.3	4.8	8.6	5.8	-	-	8.4	6.7
	(1.7, 4.5)	(2.2, 5.7)	(3.5, 5.4)	(2.9, 5.0)	(5.9, 11.3)	(3.4, 6.8)	(6.2, 12.4)	(4.0, 8.0)			(6.5, 10.1)	(5.1, 8.8)
FFMI, kg/m^2^, mean (SD)												
Males	13.8	14.3	14.1	13.0	18.8	16.6	18.4	16.4	-	-	21.8	20.2
	(1.2)	(1.3)	(1.8)	(1.3)	(1.8)	(1.5)	(2.4)	(1.8)			(1.9)	(2.3)
Females	13.2	13.2	13.8	13.7	16.8	15.2	16.8	15.2	-	-	19.8	18.6
	(1.1)	(1.0)	(1.7)	(1.8)	(1.8)	(1.3)	(2.0)	(1.5)			(2.1)	(2.8)
Waist circumference, cm, mean (SD)												
Males	61.5	63.3	62.3	59.0	85.6	76.5	82.3	75.9	83.5	72.6	90.8	86.4
	(13.9)	(9.0)	(6.0)	(3.3)	(10.5)	(7.2)	(14.0)	(7.5)	(8.9)	(6.8)	(11.2)	(10.6)
Females	61.9	63.4	65.5	63.8	88.6	77.6	89.4	78.1	84.9	71.7	90.6	84.4
	(9.3)	(8.7)	(9.1)	(7.0)	(12.8)	(9.1)	(15.3)	(9.5)	(9.7)	(7.3)	(11.4)	(11.9)

Abbreviations: BMI, body mass index; CICADA, Chronic Infections, Comorbidities and Diabetes in Africa; CLHNS, Cebu Longitudinal Health and Nutrition Survey; DIVIDS, Delhi Infant Vitamin D Supplementation tStudy; FFMI, fat-free mass index; FMI, fat mass index; HIV, human immunodeficiency virus; IQR, interquartile range; NPM, not previously malnourished; NUSTART, Nutritional Support for African Adults Starting Antiretroviral Therapy; PM, previously malnourished; SAM, Severe Acute Malnutrition; SD, standard deviation; SES, socioeconomic status; St-ATT, Starting Anti-TB Treatment.

1 CICADA, Tanzania; CLHNS, Philippines; DIVIDS, India; NUSTART, Zambia; SAM, Zambia; St-ATT, Philippines.

2 Missing data: HIV: 28 CICADA, 1 NUSTART; SES tercile: 16 DIVIDS; BMI: 7 DIVIDS, 1 NUSTART; FMI and FFMI: 7 DIVIDS, 43 CICADA, 10 NUSTART, all STATT, 60 CLHNS; Waist circumference: 7 DIVIDS, 14 CICADA, 1 NUSTART.

3 BMI for children is expected to be lower than for adults; similarly, for FMI and FFMI, which sum to BMI.

**FIGURE 1:**
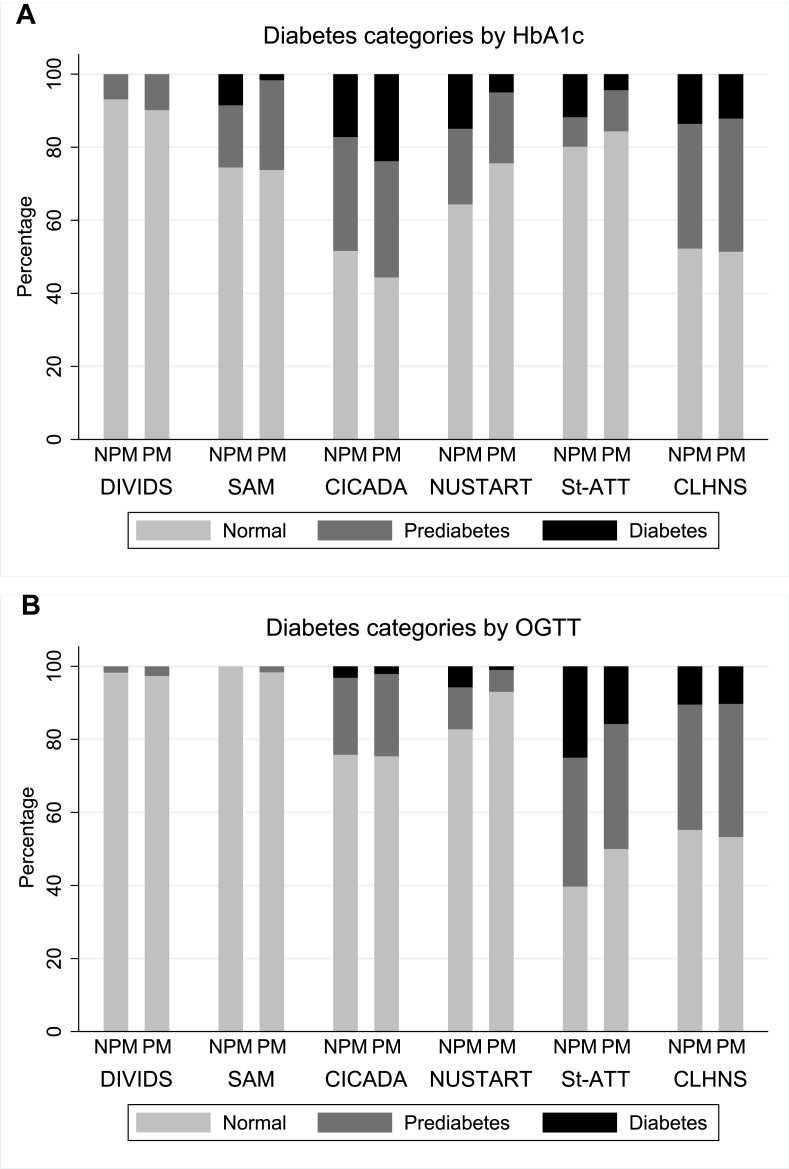
Diabetes categories by cohort and whether or not they experienced prior malnutrition (PM)^1–3^ (A) by HbA1c; (B) by oral glucose tolerance test. CICADA, Chronic Infections, Comorbidities, and Diabetes in Africa; CLHNS, Cebu Longitudinal Health and Nutrition Survey; DIVIDS, Delhi Infant Vitamin D Supplementation Study; glucose120, glucose at 120 min in an oral glucose tolerance test; HbA1c, hemoglobin A1c; NPM, not previously malnourished; NUSTART, Nutritional Support for African Adults Starting Antiretroviral Therapy; OGTT, oral glucose tolerance test; SAM, Severe Acute Malnutrition; SES, socioeconomic status; St-ATT, Starting Anti-TB Treatment. ^1^CICADA, Tanzania; CLHNS, Philippines; DIVIDS, India; NUSTART, Zambia; SAM, Zambia; St-ATT, Philippines. ^2^Missing data: HbA1c: 22 CICADA, 2 St-ATT, 7 CLHNS; OGTT: 1 DIVIDS, 2 SAM, 1 CICADA, 1 St-ATT, 1 CLHNS. ^3^Relative risk, controlling for age and sex, of PM having HbA1c ≥5.7% compared to NPM: DIVIDS, 1.28 (0.38, 4.32); SAM, 1.05 (0.53, 2.10); CICADA, 1.19 (1.05, 1.34); NUSTART, 0.39 (0.31, 0.50); St-ATT, 0.83 (0.48, 1.43); CLHNS, 0.81 (0.62, 1.05). Relative risk, controlling for age, sex, and SES, of PM having glucose120 ≥7.8 mmol/L compared to NPM: DIVIDS, 0.73 (0.07, 7.96); CICADA, 0.45 (0.37, 0.54); NUSTART, 0.11 (0.07, 0.19); St-ATT, 0.96 (0.77, 1.20); CLHNS, 0.98 (0.74, 1.30).

There was considerable heterogeneity between study cohorts in the prevalence of prediabetes and diabetes (9–51% by HbA1c, 1–56% by OGTT for combined diabetes plus prediabetes). The CICADA and CLHNS cohorts had the highest prevalence defined by HbA1c ≥5.7%, whereas St-ATT and CLHNS had the highest prevalence by OGTT, defined as glucose120 ≥7.8 mmol/L (Figure 1). Few children had prediabetes or diabetes by either test. Differences in RR, adjusted for age, sex and SES, between PM and NPM were: PM participants in CICADA had greater risk of elevated HbA1c (RR: 1.21; 95% CI: 1.07, 1.37) but lower risk of elevated glucose120 (RR: 0.61; 95% CI: 0.51, 0.73), PM in NUSTART had lower risk of elevated glucose120 (RR: 0.12; 95% CI: 0.07, 0.19), and PM in CLHNS had lower risk of elevated HbA1c (RR: 0.64; 95% CI: 0.53, 0.77).

**FIGURE 2:**
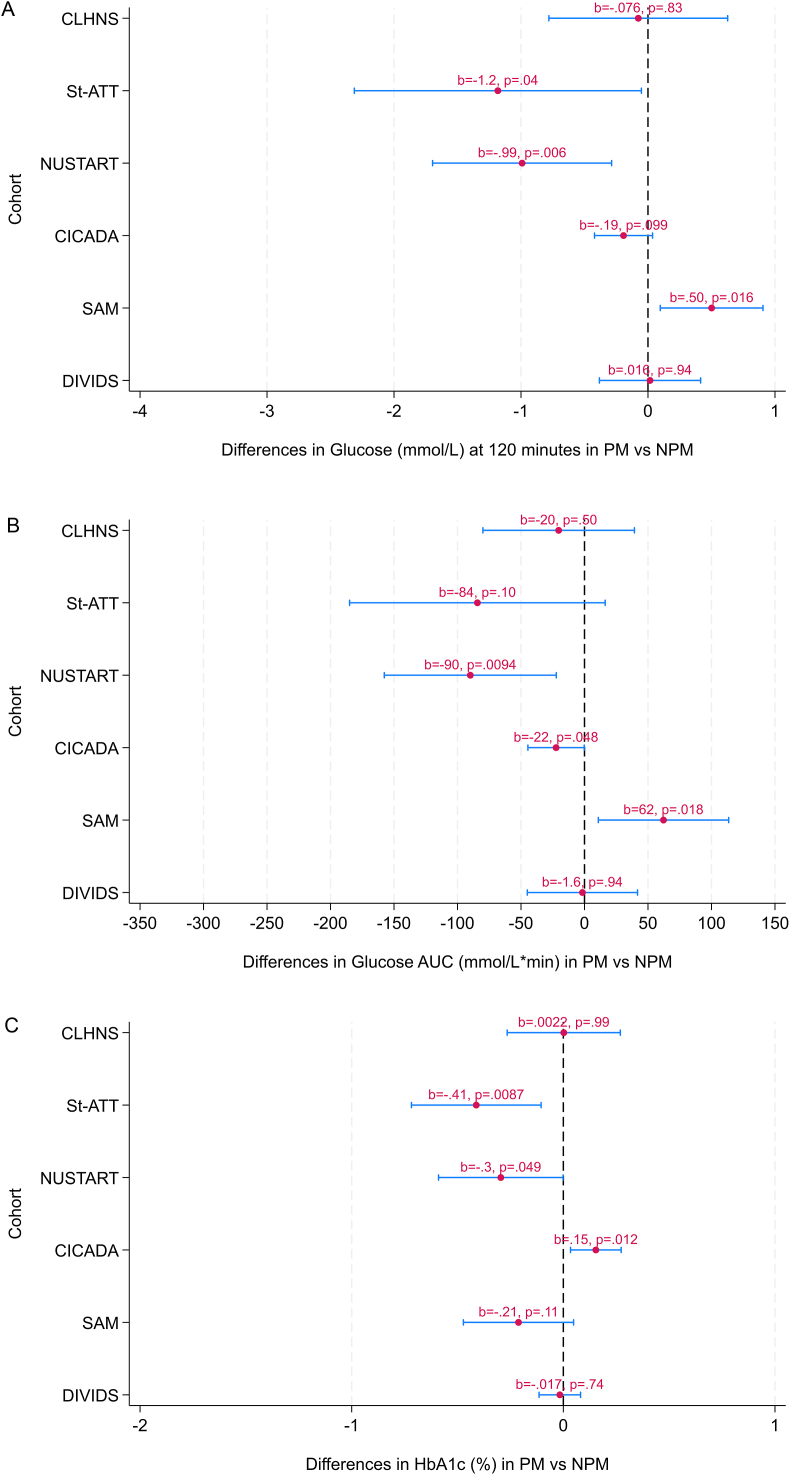
Cohort-specific adjusted mean differences between previously malnourished (PM) and not previously malnourished (NPM) for oral glucose tolerance tests (glucose at 120 min, and AUC), as well as HbA1c^1,2^. AUC, area under the curve; CI, confidence interval; CICADA, Chronic Infections, Comorbidities, and Diabetes in Africa; CLHNS, Cebu Longitudinal Health and Nutrition Survey; DIVIDS, Delhi Infant Vitamin D Supplementation Study HbA1c, hemoglobin A1c; NUSTART, Nutritional Support for African Adults Starting Antiretroviral Therapy; SAM, Severe Acute Malnutrition; SES, socioeconomic status; St-ATT, Starting Anti-TB Treatment. ^1^CICADA, Tanzania; CLHNS, Philippines; DIVIDS, India; NUSTART, Zambia; SAM, Zambia; St-ATT, Philippines.^2^Mean differences (95% CI) between PM and NPM are from linear regressions and are adjusted for age, sex, and SES.

In the continuous variable analysis (Figure 2), within cohorts, glucose120 and AUC (from plasma glucose curves during the OGTT in Supplemental Figure 2) showed similar patterns to each other: they were lower in PM than NPM in St-ATT, CICADA, and NUSTART, higher in PM than NPM in SAM (glucose120 difference 0.50 mmol/L: 95% CI: 0.10, 0.91 mmol/L), and not different between groups in DIVIDS and CLHNS. HbA1c was lower in PM in St-ATT, NUSTART, and SAM but higher in CICADA. The secondary meta-analysis (Supplemental Figure 3), which was adjusted for HIV in the African cohorts, found little difference in cohort-specific estimates and great heterogeneity across cohorts.

There was no evidence for interactions between sex and PM for either HbA1c or glucose120, among the adult cohorts studied for interactions between PM and current nutritional status (BMI, WC, and FMI), so we controlled for but did not stratify by sex. Scatterplots of glucose120 and HbA1c against current nutritional status (BMI, WC, and FMI) among adults are shown separately by cohort in Supplemental Figure 4. To facilitate comparisons across cohorts, current nutritional status markers were centered on the means for all adult cohorts: 23.2 for BMI, 83.5 cm for WC, and 6.1 for FMI. Males and females showed very similar patterns of associations of current and prior nutritional status with diabetes. Although there was some variation between cohorts and between glucose120 and HbA1c, NPM participants generally had more diabetes, indicated by values of HbA1c or glucose120 above standard cut-offs, at higher BMI, FMI, and WC, whereas PM participants also had diabetes at lower values of these. For BMI and WC, PM participant curves were more curvilinear, with a tendency to appear increased when underweight, and rising faster at higher levels. In supplementary analyses, we found that HIV or tuberculosis did not appreciably confound the associations after adjustment for age, sex, and SES (Supplemental Table 2).

**FIGURE 3:**
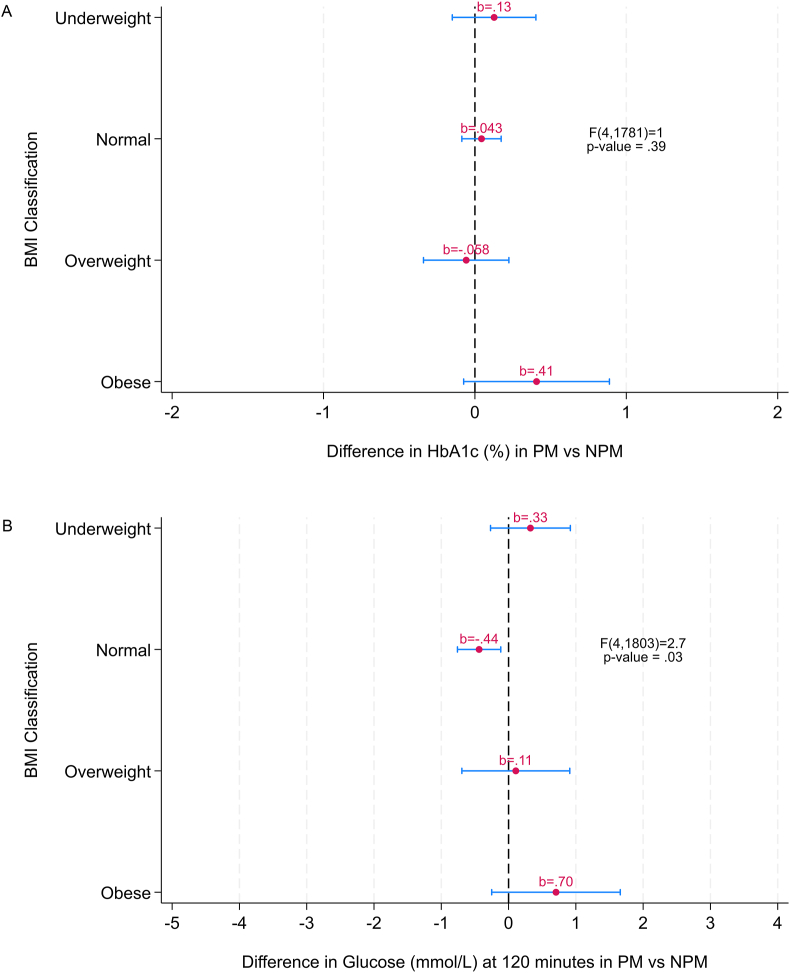
Adjusted mean differences in hemoglobin A1c and glucose at 120 min in an oral glucose tolerance test between previously malnourished (PM) and not previously malnourished (NPM) according to current nutritional status markers^1^. BMI, body mass index; HbA1c, hemoglobin A1c; SE, standard error; SES, socioeconomic status; ^1^Figures show adjusted differences between PM compared with NPM participants in HbA1c (A or glucose at 120 min in an oral glucose tolerance test (B) according to current BMI category. Analyses were by linear regression controlling for age, sex, SES, and cohort and using robust SE.

Combined cohort analyses, adjusted for age, sex, SES, and cohort (Figure 3), showed a significant interaction between PM and current BMI for glucose120 (joint *P*-value for interaction *P* = 0.03); no interaction was seen for HbA1c (joint *P*-value for interaction *P* = 0.39). Among participants currently obese, PM participants, compared to NPM, tended to have higher HbA1c (difference 0.41%; 95% CI: –0.07%, 0.89%) and glucose120 (difference 0.70 mmol/L; 95% CI: –0.25, 1.66 mmol/L). Among PM participants who continued to be underweight, there was a borderline increase, compared to NPM, in glucose120 (difference 0.33 mmol/L; 95%CI: –0.27, 0.92 mmol/L). Similar curves for individual cohorts are in Supplemental Figure 5; results are missing in some current nutrition categories because of empty cells (see Table 1).

There were no interactions for either HbA1c or glucose120 between PM and WC tertile for combined adult cohorts (Supplemental Figure 6). HbA1c was higher in PM than NPM at low FMI tercile (difference 0.25%; 95% CI: 0.09%, 0.41%). Individual cohort associations of HbA1c or glucose120 with FMI or WC tercile were variable (Supplemental Figure 5).

## Discussion

We found no overall association between PM and later risk of diabetes. However, PM may accentuate the risk of diabetes associated with adult obesity. People with prolonged undernutrition, that is, those with PM who remained underweight at the time of diabetes assessment, also had a potentially higher risk than people without PM but with current BMI <18.5. Differences in dysglycemia markers of public health importance are likely smaller than clinically relevant differences within individuals: among a large group of Chinese adults, an increase over time in mean fasting blood glucose from 4.81 mmol/L to 5.33 mmol/L translated into a 4.4% increase in prevalence of elevated glucose [26]; the size of the difference was similar to the differences we found between PM and NPM for glucose120 in individuals with obesity and in continued underweight participants.

Although some PM participants were overweight or obese at the time of SAMPA investigation and had increased diabetes risk, their mean BMI remained lower than that of the NPM for all cohorts except DIVIDS. This may partly explain the lack of crude associations between PM and diabetes risk in the overall cohorts, with increased risk being driven by weight gain following malnutrition. In addition, since we used internal terciles for WC and FMI, rather than external cut-offs as for BMI, the highest terciles may have included people who did not have excess overall or central fat.

There was considerable heterogeneity among cohorts in how PM was associated with later glucose metabolism. The original hypothesis of the SAMPA study was that PM induces an irreversible decrement in functional endocrine mass within the pancreas at the time of malnutrition, so that the pancreas’ metabolic capacity is lower. There may be a signal in line with this possibility because there was a tendency for consistently underweight adult PM participants to have borderline abnormal glucose metabolism. With regards to the capacity-load hypothesis, our analyses suggest excess weight gain may unmask earlier pancreatic damage, resulting in elevated diabetes markers among PM participants with obesity.

Previous CICADA cohort analyses found lower insulin production during an OGTT in males, but not females, with PM [6], suggesting that pancreatic endocrine reserve capacity is indeed less in people with PM. In SAMPA, although we did find higher HbA1c and glucose120 overall among adult participants with obesity, the individual cohort analyses suggest this was driven largely by CLHNS, which was the cohort with the longest duration of follow-up since childhood PM and the highest prevalence of obesity. Furthermore, we used the same BMI cut-offs for all cohorts, so functional obesity prevalence would have been higher in CLHNS if we had used lower Asian BMI cut-offs. Participants in DIVIDS and SAM also had PM in early life, but they were still children and had little obesity or diabetes; however, PM, compared to NPM, participants in SAM had higher glucose120 and area under the glucose curve.

There were multiple underlying differences between cohorts in addition to age and proximal cause of PM. For example, in the 4 countries represented, there were major differences in diet, in both intensity of exposure and type of infections, and in health care available. The Indian and Zambian cohorts were fully urban, whereas the Tanzanian and Filipino cohorts also included participants from peri-urban and rural areas. It is important to note that, of the cohorts, only CLHNS was representative of the local population at the time of birth cohort recruitment and, due to attrition and sampling for SAMPA, was no longer representative. The other cohorts were selected for reasons related to the original study aims. Most PM in the CICADA, NUSTART, and St-ATT cohorts was associated with HIV and/or tuberculosis infection, but within-cohort analyses, adjusting for HIV did not change within-cohort results (Supplemental Table 2). Because the cause of malnutrition and time since PM are collinear with the cohort, our combined analyses adjusting for the cohort accounted for confounding by the cause of PM. Furthermore, wasting malnutrition carries a high mortality risk, which may result in survival bias. For example, for the NUSTART cohort and the NUSTART participants in the CICADA cohort, males and participants with initially lower BMI were at increased risk of early death within 12 wk of starting antiretroviral therapy [19].

There was a larger deficit in FMI for females than males in the African cohorts, but we found no interaction between sex and PM for associations with HbA1c or glucose120. However, since there were variable sex differences in the meta-analysis of wasting malnutrition and later diabetes [3], sex differences should be considered in all studies of long-term follow-up of malnourished people.

We considered glucose120 as the primary diagnostic marker for diabetes because of the challenges posed by both HbA1c and fasting plasma glucose in specific populations. Reports from sub-Saharan Africa and Southeast Asia suggest hemoglobin variants and hemoglobinopathies to be common, likely affecting the concentrations of HbA1c [27,28]. Furthermore, fasting plasma glucose often presents variability that does not correspond with postprandial glucose tolerance, complicating its use as a reliable marker for diabetes diagnosis [14]. We decided not to combine the different diagnostic measures to avoid conflating markers that may not be consistent across diverse cohorts.

Strengths of the study include its large multi-country cohort and the fact that the earlier nutritional status of both PM and NPM participants was assessed with research-quality anthropometry. This is in contrast to otherwise excellent work following up the mid-twentieth-century Chinese famine, in which exposure to famine and thus likely wasting malnutrition was inferred from the date and location of birth [5]. There is likely to be variation within a region and between households as to the severity of individual nutritional deprivation during a famine; there is evidence from the Dutch Hunger Winter that higher perceived severity of nutritional stress was associated with a greater risk of later type 2 diabetes [24]. Our study also has some limitations. Although a causal association is plausible, whereby reduced pancreas capacity after PM interacts with increased metabolic load in obesity to increase the risk of diabetes, it cannot be proven due to the study design. However, this inability to prove causality is common to all research in the field and our cohort study design, with objectively measured PM preceding diabetes assessment by many years in some cases, provides better evidence than contemporary cross-sectional designs, and the heterogeneity among adult cohorts gives us a pointer as to when to expect an impact on glucose metabolism, i.e., many years later and possibly only if people gain weight. Secondly, we have no knowledge of factors associated with malnutrition before cohort recruitment, e.g., birthweight of the SAM cohort, or of the nutritional status in childhood of participants from CICADA, NUSTART, and St-ATT, since they were first recruited as adults, so some people in the NPM group with consistently normal BMI could have experienced childhood wasting. Related to this, there may be unmeasured confounding, especially given the limited potential confounders for which data were available across cohorts. Thirdly, for some participants in CICADA and St-ATT cohorts for which diabetes assessment was an aim, diabetes was diagnosed at initial recruitment when PM was assessed, so we cannot determine causality in those cases. Fourthly, although our cohorts included people from several countries and a wide range of environments and causes of PM, none of the cohorts was a random sample of their population, which limits the ability to generalize. Fifthly, we had low statistical power for interaction analyses. Finally, as in all studies of long-term outcomes after acute malnutrition, there is a survivor bias.

In summary, people who previously suffered from wasting should gain at least some weight, not only for immediate benefits but also since continued underweight was associated with higher glucose120. However, clinicians and public health managers should promote education and interventions to prevent excess weight gain since this appears associated with even greater risk of diabetes in people with PM than in the general population. Our results also provide further justification for initiatives to avoid children and adults from becoming malnourished in the first place.

## Author contributions

The authors’ responsibilities were as follows– SEC, PD, DF-J, PK, LK, RK, RK-M, NL, DN, GP, JAMS, JAS, GT-K, SF: designed the study, and obtained funding; DPM, SA, SF: verified data; DPM: conducted statistical analyses with input from SA, RD, RK, NL, DN JAMS, SF; DPM, SA: produced the figures; SF: drafted the manuscript, and all other authors reviewed and edited the text; SF: is the guarantor of this work and, as such, had full access to all the data in the study and takes responsibility for the integrity of the data and the accuracy of the data analysis; and all authors: read and approved the final manuscript.

## Data availability

Upon application to the principal investigator, data will be made available for bona fide research adhering to ethical guidelines by which the study was conducted.

## Funding

The Severe Acute Malnutrition - the Role of the Pancreas study was supported by funding from the Medical Research Council, United Kingdom (reference number MR/V000578/1). The funder had no role in the study design, in the collection, analysis, or interpretation of the data, or in the decision to submit the paper for publication.

## Conflict of interest

JAMS has attended a Mogrify Scientific Advisory Board under a consultancy agreement with Newcastle University. All other authors report no conflicts of interest.
