# Supplementary material for: Associations of prior wasting malnutrition with later indicators of glucose tolerance across 4 countries in Africa and Asia: The Severe Acute Malnutrition - the Role of the Pancreas (SAMPA) study
Source: Am J Clin Nutr. 2025 Aug 5;122(4):986–96. doi: 10.1016/j.ajcnut.2025.07.032 (PMC12674020; doi:10.1016/j.ajcnut.2025.07.032)
Supplement: Supplementary file 1 [file mmc1.docx]

**Supplemental material**

|  | **Page** |
| --- | --- |
| 1. Supplemental Table 1. Comparison of original cohort members included and not included in SAMPA | 2 |
| 2. Supplemental Table 2. Influence of HIV and tuberculosis infection on the difference in HbA1c and glucose at 120 minutes in an oral glucose tolerance test between African adult participants with prior malnutrition and no prior malnutrition | 7 |
| 3. Supplemental Figure 1. Flow charts for study cohorts | 9 |
| 4. Supplemental Figure 2. Plasma glucose during an oral glucose tolerance test for each cohort, divided by sex and whether or not participants experienced prior malnutrition | 15 |
| 5. Supplemental Figure 3. A meta-analysis of cohort-specific adjusted mean differences between previously malnourished and not previously malnourished for oral glucose tolerance tests (glucose at 120 minutes), and HbA1c | 17 |
| 6. Supplemental Figure 4. Scatter plots of diabetes indicators by prior malnutrition and current nutritional status markers among adult cohorts | 18 |
| 7. Supplemental Figure 5. Interactions between current nutritional status and prior malnutrition for associations with HbA1c and glucose at 120 minutes in an oral glucose tolerance test in cohorts separately | 24 |
| 8. Supplemental Figure 6. Adjusted mean differences in HbA1c and glucose at 120 minutes in an oral glucose tolerance test between previously malnourished and not previously malnourished adult participants according to current waist circumference and fat mass index | 31 |
| 9. References | 33 |

**Supplemental Table 1. Comparison of original cohort members included and not included in SAMPA^1,2,3^**

|  | Not included in SAMPA | Included in SAMPA | P |
| --- | --- | --- | --- |
| DIVIDS ^4^ | | | |
| N | 1817 | 264 |  |
| % Females | 973 (53.6%) | 134 (50.8%) | 0.40 |
| Birth weight (kg) | 2.2 (0.17) | 2.2 (0.16) | 0.22 |
| Age at inclusion (years), mean (SD) | 0 (newborn) | 0 (newborn) | - |
| SES, # (%) |  |  | <0.001 |
| Lowest | 388 (21.4%) | 28 (10.7%) |  |
| Low | 374 (20.6%) | 42 (16.0%) |  |
| Middle | 363 (20.0%) | 53 (20.2%) |  |
| High | 348 (19.1%) | 68 (26.0%) |  |
| Highest | 344 (18.9%) | 71 (27.1%) |  |
| Mothers’ education, # (%) |  |  | 0.001 |
| No education | 417 (23.0%) | 28 (10.7%) |  |
| Primary or less | 279 (15.3%) | 43 (16.3%) |  |
| Middle | 527 (29.0%) | 90 (34.1%) |  |
| Secondary | 474 (26.1%) | 90 (34.1%) |  |
| College/university | 120 (6.6%) | 13 (4.9%) |  |
| Father’s occupation |  |  |  |
| Unemployed | 43 (2.4%) | 11 (4.2%) |  |
| Unskilled | 133 (7.3%) | 16 (6.1%) |  |
| Semi-skilled | 417 (23.0%) | 55 (20.8%) | 0.81 |
| Skilled | 1,039 (57.2%) | 145 (54.9%) |  |
| Self-employed | 165 (9.1%) | 33 (12.5%) |  |
| Professional | 20 (1.1%) | 4 (1.5%) |  |
| SAM | | | |
| N | 77 | 108 |  |
| % Females | 36 (46.8%) | 50 (46.3%) | 0.35 |
| BMIZ, mean (SD) | -0.61 (1.1) | -0.73 (1.0) | 0.42 |
| Age at inclusion (years), mean (SD) | 9.3 (1.7) | 9.0 (1.4) | 0.12 |
| SES, # (%) |  |  | 0.83 |
| Low | 58 (75.3%) | 83 (76.9%) |  |
| Middle | 8 (10.4%) | 10 (9.2%) |  |
| High | 11 (14.3%) | 15 (13.9%) |  |
| Mothers’ education, # (%) |  |  | 0.68 |
| Primary or less | 33 (42.9%) | 25 (41.0%) |  |
| Secondary | 35 (45.4%) | 31 (50.8%) |  |
| College/university | 7 (9.1%) | 4 (6.6%) |  |
| Missing | 2 (2.6%) | 1 (1.6%) |  |
| Father’s occupation, # (%) |  |  | 0.95 |
| Unemployed | 2 (2.6%) | 1 (0.9%) |  |
| Salaried | 36 (46.8%) | 50 (46.3%) |  |
| Self-employed | 29 (37.7%) | 41 (38.0%) |  |
| Other | 10 (13.0%) | 16 (14.8%) |  |
| CICADA | | | |
| N (Total) | 973 | 974 |  |
| a) TB-NUT group (2006-2009) |  |  |  |
| N | 166 | 284 |  |
| % Females | 81 (48.8%) | 142 (50.0%) | 0.81 |
| BMI (kg/m^2^), mean (SD) | 20.8 (4.3) | 20.4 (4.0) | 0.31 |
| Age at inclusion (years), mean (SD) | 38.9 (13.1) | 37.1 (11.8) | 0.13 |
| SES, # (%) |  |  | 0.40 |
| Lowest | 36 (21.7%) | 66 (23.2%) |  |
| Low | 24 (14.4%) | 50.0 (17.6%) |  |
| Middle | 30 (18.1%) | 55 (19.4%) |  |
| High | 37 (22.3%) | 47 (16.6%) |  |
| Highest | 39 (23.5%) | 66 (23.2%) |  |
| Education, # (%) |  |  | 0.62 |
| No education | 28 (16.9%) | 49 (17.3%) |  |
| Primary or less | 108 (65.1%) | 191 (67.3%) |  |
| Secondary | 22 (13.3%) | 31 (10.9%) |  |
| College/university | 2 (1.2%) | 3 (1.1%) |  |
| Vocational | 6 (3.6%) | 10 (3.5%) |  |
| Occupation, # (%) |  |  | 0.050 |
| Unemployed | 37 (22.3%) | 26 (9.2%) |  |
| Salaried | 22 (13.2%) | 37 (13.0%) |  |
| Self-employed | 107 (64.5%) | 221 (90.8%) |  |
| Others | 0 | 0 |  |
| b) NUSTART, Tanzania group (2016-2017) |  |  |  |
| N | 75 | 133 |  |
| % Females | 46 (61.3%) | 85 (64.4%) | 0.71 |
| BMI (kg/m^2^), mean (SD) | 16.8 (1.3) | 16.7 (1.1) | 0.54 |
| Age at inclusion (years), mean (SD) | 38.5 (9.0) | 39.3 (9.7) | 0.54 |
| SES, # (%) |  |  | 0.20 |
| Lowest | 29 (38.7%) | 48 (36.4%) |  |
| Low | 26 (34.7%) | 31 (23.5%) |  |
| Middle | 7 (9.3%) | 24 (18.2%) |  |
| High | 11 (14.7%) | 13 (9.9%) |  |
| Highest | 2 (2.7%) | 16 (12.1%) |  |
| Education, # (%) |  |  | 0.13 |
| No education | 27 (36.0%) | 37 (28.0%) |  |
| Primary or less | 45 (60.0%) | 83 (62.9%) |  |
| Secondary | 3 (4.0%) | 12 (9.1%) |  |
| Occupation, # (%) |  |  | 0.20 |
| Unemployed | 14 (18.7%) | 11 (8.3%) |  |
| Salaried | 7 (1.3%) | 15 (11.4%) |  |
| Self-employed | 54 (72%) | 106 (80.3%) |  |
| Others | 0 | 0 |  |
| c) Recruited at CICADA baseline (2018-2019 ) |  |  |  |
| N | 732 | 557 |  |
| % Females | 464 (63.4%) | 339 (60.9%) | 0.35 |
| BMI (kg/m^2^), mean (SD) | 21.6 (4.4) | 22.3 (4.7) | 0.005 |
| Age at inclusion (years), mean (SD) | 37.0 (11.5) | 39.4 (10.5) | <0.001 |
| SES, # (%) |  |  | 0.78 |
| Lowest | 112 (15.4%) | 98 (17.6%) |  |
| Low | 153 (21.0%) | 108 (18.7%) |  |
| Middle | 153 (21.0%) | 120 (21.6%) |  |
| High | 162 (22.2%) | 118 (21.2%) |  |
| Highest | 149 (20.4%) | 116 (20.9%) |  |
| Education, # (%) |  |  | 0.47 |
| No education | 112 (15.4%) | 66 (11.9%) |  |
| Primary or less | 495 (67.9%) | 404 (72.7%) |  |
| Secondary | 104 (14.3%) | 73 (13.1%) |  |
| College/university | 4 (0.6%) | 1 (0.2%) |  |
| Vocational | 14 (1.9%) | 12 (2.2%) |  |
| Occupation, # (%) |  |  | 0.88 |
| Unemployed | 134 (18.4%) | 69 (12.4%) |  |
| Salaried | 112 (15.4%) | 75 (13.5%) |  |
| Self-employed | 482 (66.1%) | 411 (73.9%) |  |
| Others | 1 (0.1%) | 1 (0.2%) |  |
| NUSTART, Zambia | | | |
| N | 912 | 201 |  |
| Females, # (%) | 402 (44.1%) | 111 (55.8%) | 0.003 |
| BMI (kg/m^2^), mean (SD) | 16.3 (1.4) | 16.9 (1.6) | <0.001 |
| Age at inclusion (years), mean (SD) | 34.3 (9.1) | 34.9 (7.6) | 0.42 |
| SES, # (%) |  |  | 0.04 |
| Lowest | 195 (21.4%) | 28 (14.1%) |  |
| Low | 182 (20.0%) | 42 (21.1%) |  |
| Middle | 192 (21.1%) | 38 (19.1%) |  |
| High | 165 (18.1%) | 49 (24.6%) |  |
| Highest | 178 (19.5%) | 42 (21.1%) |  |
| Education, # (%) |  |  | 0.26 |
| No education | 131 (14.4%) | 18 (9.1%) |  |
| Primary or less | 464 (50.9%) | 109 (54.8%) |  |
| Secondary | 281 (30.8%) | 64 (32.2%) |  |
| College/university | 36 (3.9%) | 8 (4.0%) |  |
| Occupation, # (%) |  |  | 0.25 |
| Salaried | 198 (21.7%) | 49 (24.6%) |  |
| Self employed | 273 (29.9%) | 65 (32.7%) |  |
| Housewife | 117 (12.8%) | 31 (15.6%) |  |
| Student | 10 (1.1%) | 2 (1.0%) |  |
| Unemployed | 314 (34.4%) | 52 (26.1%) |  |
| St-ATT ^5^ | | | |
| N | 443 | 251 |  |
| % Females | 119 (26.9%) | 74 (29.5%) | 0.46 |
| BMI (kg/m^2^), mean (SD) | 19.4 (3.2) | 19.4 (3.3) | 0.99 |
| Age at inclusion (years), mean (SD) | 44.5 (17.6) | 46.2 (15.5) | 0.21 |
| Family income (PHP) # (%) |  |  | 0.003 |
| Less than 5,000 | 167 (37.8%) | 129 (51.4%) |  |
| 5000 - 9999 | 121 (27.4%) | 69 (27.5%) |  |
| 10,000 - 14,999 | 102 (23.1%) | 35 (13.9%) |  |
| 15,000-19,999 | 18 (4.1%) | 8 (3.2%) |  |
| 20,000 or more | 22 (5.0%) | 5 (2.0%) |  |
| Don't know | 12 (2.7%) | 5 (2.0%) |  |
| Education, # (%) |  |  | 0.051 |
| Primary or less | 135 (30.5%) | 90 (35.9%) |  |
| Secondary | 195 (44.1%) | 120 (47.8%) |  |
| Tertiary school | 80 (18.1%) | 29 (11.6%) |  |
| Vocational | 32 (7.2%) | 12 (4.8%) |  |
| Occupation, # (%) |  |  | 0.24 |
| Unemployed | 273 (61.8%) | 166 (60.4%) |  |
| Daily wager | 85 (19.2%) | 72 (26.2%) |  |
| Salaried | 2 (0.5%) | 0 |  |
| Other | 82 (18.6%) | 37 (13.4%) |  |
| CLHNS | | | |
| N | 2772 | 308 |  |
| % Females | 1337 (48.2%) | 111 (36.0%) | <0.001 |
| WHZ, mean (SD) | -0.74 (1.3) | -0.86 (1.5) | 0.15 |
| Age at inclusion (years), mean (SD) | 0 (Newborn) | 0 (Newborn) | - |
| SES, # (%) |  |  | 0.13 |
| Lowest | 638 (23.0%) | 54 (17.5%) |  |
| Low | 492 (17.8%) | 53 (17.2%) |  |
| Middle | 878 (31.7%) | 114 (37.0%) |  |
| High | 214 (7.7%) | 29 (9.4%) |  |
| Highest | 550 (19.8%) | 58 (18.8%) |  |
| Education, # (%) |  |  | 0.006 |
| No education | 53 (1.9%) | 5 (1.6%) |  |
| Primary or less | 1441 (52.0%) | 185 (60.1%) |  |
| Secondary | 913 (32.9%) | 93 (30.2%) |  |
| College/university | 365 (13.2%) | 25 (8.1%) |  |

^1^ BMI, body mass index; CICADA, Chronic Infections, Co-morbidities and Diabetes in Africa; CLHNS; Cebu Longitudinal Health and Nutrition Survey; DIVIDS, Delhi Infant Vitamin D Supplementation Trial; NUSTART, Nutritional Support for African Adults Starting Anti-retroviral Therapy; PHP, Philippine Peso; SAM, Severe Acute Malnutrition; SD, standard deviation; SES, socioeconomic status of participants in the original cohort; St-ATT; Starting Anti-TB Treatment; TB-NUT, Nutrition, Diabetes, and Pulmonary Tuberculosis; WHZ, weight-for-length Z score

^2^ Analyses were by linear regression for continuous variables, chi-square test and ordered logistic regression for categorical variables.

^3^ Not previously malnourished controls were recruited to DIVIDS and NUSTART Lusaka only for SAMPA, so the ‘included in SAMPA’ column refers only to the previously malnourished participants.

^4^ In the DIVIDS primary trial analysis, two participants who were excluded because of congenital abnormalities are included in SAMPA so the total sample size is 2081.

^5^ In St-ATT, ‘not included in SAMPA’ does not include participants from the Manila site since we knew this highly mobile population would be very hard to trace and thus were purposefully excluded during SAMPA recruitment.

**Supplemental Table 2. Influence of HIV and tuberculosis infection on the difference in HbA1c and in glucose at 120 minutes in an oral glucose tolerance test between African adult participants with prior malnutrition and no prior malnutrition^1,2^**

Hba1c

Cohort	Model	BMI	Coefficient	SE	P-	Lower	Upper

value	limit	limit

CICADA	Base model	Underweight	0.07	0.21	0.74	-0.34	0.48

(age, sex,	Normal	0.22	0.074	0.0027	0.077	0.37

SES, BMI)	Overweight	0.12	0.20	0.54	-0.26	0.51

Obese	0.75	0.42	0.074	-0.072	1.60

Base model	Underweight	0.055	0.21	0.79	-0.36	0.47

+ HIV	Normal	0.20	0.075	0.0067	0.057	0.35

Overweight	0.10	0.20	0.60	-0.28	0.49

Obese	0.75	0.43	0.079	-0.087	1.60

Base model	Underweight	0.04	0.20	0.84	-0.35	0.43

+ HIV + TB	Normal	0.17	0.076	0.028	0.018	0.32

Overweight	0.051	0.19	0.79	-0.32	0.43

Obese	0.78	0.42	0.066	-0.051	1.60

NUSTART	Base model	Underweight	.	.	.	.	.

(age, sex,	Normal	-0.17	0.24	0.49	-0.65	0.31

SES, BMI)	Overweight	-0.14	0.29	0.62	-0.72	0.43

Obese	-0.59	0.25	0.018	-1.10	-0.10

Base model	Underweight	.	.	.	.	.

+ HIV	Normal	-0.15	0.19	0.43	-0.54	0.23

Overweight	-0.12	0.30	0.68	-0.71	0.46

Obese	-0.56	0.35	0.11	-1.30	0.13

Base model	Underweight	.	.	.	.	.

+ HIV + TB	Normal	-0.16	0.21	0.44	-0.57	0.25

Overweight	-0.13	0.30	0.67	-0.72	0.46

Obese	-0.58	0.36	0.11	-1.30	0.14

Glucose at 120 minutes

Cohort	Model	BMI	Coefficient	SE	P-	Lower	Upper

value	limit	limit

CICADA	Base model	Underweight	-0.19	0.28	0.49	-0.74	0.35

(age, sex,	Normal	-0.0075	0.14	1.00	-0.28	0.28

SES, BMI)	Overweight	-0.11	0.48	0.82	-1.00	0.83

Obese	-0.62	0.40	0.12	-1.40	0.17

Base model	Underweight	-0.14	0.28	0.61	-0.70	0.41

+ HIV	Normal	0.064	0.14	0.64	-0.21	0.34

Overweight	-0.032	0.48	0.95	-0.97	0.90

Obese	-0.6	0.40	0.14	-1.40	0.19

Base model	Underweight	-0.18	0.27	0.51	-0.71	0.36

+ HIV + TB	Normal	-0.012	0.14	0.93	-0.28	0.25

Overweight	-0.14	0.48	0.77	-1.1	0.80

Obese	-0.54	0.42	0.19	-1.4	0.28

NUSTART	Base model	Underweight	.	.	.	.	.

(age, sex,	Normal	-0.87	0.52	0.091	-1.9	0.14

SES, BMI)	Overweight	-0.57	0.81	0.48	-2.2	1.00

Obese	-0.81	0.68	0.24	-2.2	0.54

Base model	Underweight	.	.	.	.	.

+ HIV	Normal	-0.45	0.40	0.26	-1.2	0.34

Overweight	0.024	0.76	0.97	-1.5	1.50

Obese	0.05	0.99	0.96	-1.9	2.00

Base model	Underweight	.	.	.	.	.

+ HIV + TB	Normal	-0.35	0.40	0.39	-1.1	0.44

Overweight	0.12	0.76	0.87	-1.4	1.60

Obese	0.29	0.99	0.77	-1.7	2.20

^1^ CICADA, Chronic Infections, Co-morbidities and Diabetes in Africa; NUSTART, Nutritional Support for African Adults Starting Antiretroviral Therapy; TB, tuberculosis; TB-NUT, Nutrition, Diabetes, and Pulmonary Tuberculosis

^2^ Differences by linear regression controlling for age, sex, SES, and BMI

**Supplemental Figure 1. Flow charts for study cohorts^1,2^**

**A. DIVIDS (Delhi Infant Vitamin D Supplementation) Trial,** **India**

Initial recruitment (2007-2010)

n=2079

Total DIVIDS Recruitment

n=322 (58 NPM, 264 PM)

Logistic issues so not invited, n=409

refused, n=3; other, n=20

Died, n=55; refused, n=81; other (unable to contact, migrated, withdrew, insufficient funds to recruit) n =1276

Potentially available for SAMPA

n=754

n=87**,** Normal birth weight

n=667**,** Low birth weight

B. **SAM (Severe Acute Malnutrition), Lusaka, Zambia**

Initial recruitment (2010-2014)

n=2034

Refused, n=30; other (unable to contact, moved, ineligible, funding limitations), n=1904

Total SAM Recruitment

n=108 (47 NPM, 61 PM)

Not-previously malnourished neighbourhood controls, n=85

Potentially available to SAMPA

n=100, previously malnourished

Refused, n=17; unable to contact, n=30; relocated, n=30

C. **CICADA (Chronic Infections, Co-morbidities and Diabetes in Africa), Mwanza, Tanzania**

Initial recruitment

Potentially available for SAMPA study

n=1412

Died, n=149; refused, n=70;

other (not reachable, moved), n=316

Died, n=304; refused, n=2; other (moved or not found), n=193

Died, n=47; refused, n=34; unable to contact, n=235; relocated, n=122

Total CICADA Recruitment

n=974 (637 NPM, 337 PM)

Died, n=291; refused, n=18; other (moved or not found), n=1247

NUSTART cohort, n=707

(2011-2013)

TB-NUT Cohort, n=2006

(2006-2009)

CICADA Cohort, n=1947

(2016-2019)

D. **NUSTART (Nutritional Support for Africans Starting Antiretroviral Therapy), Lusaka, Zambia**

Died, n=20; refused, n=35; unable to contact, n=211; relocated, n= 63

Invited to join SAMPA

n=530

Died, n=224; withdrew, n=142

Not previously malnourished controls, n=87

Total NUSTART Recruitment

n=288 (87 NPM, 201 PM)

Potentially available to SAMPA

n=747

Initial recruitment

HIV-infected with low BMI when starting ART, n=1113

E. **St-ATT (Starting Anti-TB Treatment) Cohort, Philippines**

Total St-ATT Recruitment

n=251 (136 NPM, 115 PM)

Died, n=26; refused, n=66; unable to contact, n=56; relocated, n=24; diabetes history, n=10; other, n=2

346346

Died, n=17; refused, n=91; unable to contact, n=87; relocated, n=40; diabetes history, n=14; other, n=1

No SAMPA enrolment from Manila site

Initial recruitment

n=902

Manila

n=184

Cebu

n=346

Potentially available to SAMPA

n=332

Died, n=14

Negros

n=372

Potentially available to SAMPA

n=354

Total Negros recruitment

n=170

Total Cebu recruitment

n=81

Died, n=18

F. **CLHNS (Cebu Longitudinal Health and Nutrition Survey), Philippines**

Initial recruitment (1983-84)

n = 3080

Died, n= 287; migrated out of Cebu, n= 1,257; other, n=210

Potentially available to SAMPA

n=1326

Refused, n=121; died, n=9

relocated, n=22; other, n=16

Total CLHNS Recruitment

n=308 (201 NPM, 107 PM)

Invited to join SAMPA

n=476

(malnourished in first 2y=143)

^1^ Details of participant flow charts between initial recruitment and potential availability to SAMPA are published.(7)

^2^ NPM, not previously malnourished; PM, prior wasting malnutrition

**Supplemental Figure 2. Plasma glucose during an oral glucose tolerance test for each cohort, divided by sex and whether or not participants experienced prior malnutrition^1,2^**

^1^ Y axis scale differs for St-ATT from other cohorts due to the higher glucose levels.

^2^ CICADA, Chronic Infections, Co-morbidities and Diabetes in Africa, Tanzania; CLHNS, Cebu Longitudinal Health and Nutrition Survey, Philippines; DIVIDS, Delhi Infant Vitamin D Supplementation Trial, India; NPM, not previously malnourished; NUSTART, Nutritional Support for African Adults Starting Anti-retroviral Therapy, Zambia; PM, previously malnourished; SAM, Severe Acute Malnutrition, Zambia; St-ATT, Starting Ant-TB Treatment, Philippines

**Supplemental Figure 3. A meta-analysis of cohort-specific adjusted mean differences between previously malnourished and not previously malnourished for oral glucose tolerance tests (glucose at 120 minutes), and HbA1c**

^1^DIVIDS, Delhi Infant Vitamin D Supplementation Trial, India; SAM, Severe Acute Malnutrition, Zambia; CICADA, Chronic Infections, Co-morbidities and Diabetes in Africa, Tanzania; NUSTART, Nutritional Support for African Adults Starting Anti-retroviral Therapy, Zambia; St-ATT, Starting Ant-TB Treatment, Philippines; CLHNS, Cebu Longitudinal Health and Nutrition Survey, Philippines

^2^ Mean differences (95% CI) between PM and NPM are from linear regression. Models for DIVIDS, St-ATT and CLHNS were adjusted for age, sex and socioeconomic status (SES), while models for SAM, CICADA and NUSTART additionally adjusted for HIV status.

**B. Hba1c**

**A. Glucose**

**Supplemental Figure 4. Scatter plots of diabetes indicators by prior malnutrition and current nutritional status markers among adult cohorts^1,2^**

^1^ Figures show glucose at 120 minutes in an oral glucose tolerance test (glucose 120) or haemoglobin A1c (HbA1c) according to prior wasting malnutrition and current nutritional status indicators: body mass index (BMI), waist circumference, and fat mass index (FMI). To facilitate comparisons across cohorts with varying levels of nutritional status, values for current nutritional status were centred on the values for all adult cohorts together: 23.2 kg/m^2^ for BMI, 83.5 cm for waist circumference, and 6.1 kg/m^2^ for FMI. Curves are from quadratic regressions for PM and NPM separately. Horizontal dotted lines indicate the cut-offs for prediabetes and diabetes: for glucose120, the cut-offs are 7.8 mmol/L (prediabetes) and 11.1 mmol/L (diabetes); for HbA1c, the cut-offs are 5.7% (prediabetes) and 6.5% (diabetes).

^2^ CICADA, Chronic Infections, Co-morbidities and Diabetes in Africa, Tanzania; CLHNS, Cebu Longitudinal Health and Nutrition Survey, Philippines; DIVIDS, Delhi Infant Vitamin D Supplementation Trial, India; NPM, not previously malnourished; NUSTART, Nutritional Support for African Adults Starting Anti-retroviral Therapy, Zambia; PM, previously malnourished; SAM, Severe Acute Malnutrition, Zambia; St-ATT, Starting Ant-TB Treatment, Philippines

**Supplemental Figure 5. Interactions between current nutritional status and prior malnutrition for associations with HbA1c and glucose at 120 minutes in an oral glucose tolerance test in cohorts separately^1,2^**

**A**

**Difference in HbA1c in PM vs NPM, by BMI categories**

**B**

**Difference in Glucose at 120 minutes in PM vs NPM, by BMI categories**

**C**

**Difference in HbA1c in PM vs NPM, by waist circumference tercile**

**D**

**Difference in Glucose at 120 minutes in PM vs NPM, by waist circumference tercile**

**E**

**Difference in HbA1c in PM vs NPM, by FMI tercile**

**F**

**Difference in Glucose at 120 minutes in PM vs NPM, by FMI tercile**

^1^ Figures show adjusted differences between PM versus NPM participants in HbA1c (A, C, E) or glucose at 120 minutes in an oral glucose tolerance test (B, D, F) according to current body mass index category (A, B), or terciles of waist circumference (C, D) or fat mass index (E, F). Analyses were by linear regression controlling for age and sex and, for the combined analyses, cohort as well, and used robust standard errors. St-ATT participants were not included in fat mass index analyses because of lack of data.

^2^ CICADA, Chronic Infections, Co-morbidities and Diabetes in Africa, Tanzania; CLHNS, Cebu Longitudinal Health and Nutrition Survey, Philippines; DIVIDS, Delhi Infant Vitamin D Supplementation Trial, India; FMI, fat mass index; NPM, not previously malnourished; NUSTART, Nutritional Support for African Adults Starting Anti-retroviral Therapy, Zambia; PM, previously malnourished; SAM, Severe Acute Malnutrition, Zambia; St-ATT, Starting Ant-TB Treatment, Philippines

**Supplemental Figure 6.** **Adjusted mean differences in HbA1c and glucose at 120 minutes in an oral glucose tolerance test between previously malnourished and not previously malnourished adult participants according to current waist circumference and fat mass index^1,2^**

**A**

**B**

**C**

**D**

^1^ Figures show adjusted differences between PM versus NPM participants in HbA1c (A, C) or glucose at 120 minutes in an oral glucose tolerance test (B, D) according to current terciles of waist circumference (A, B) or fat mass index (C, D). Analyses were by linear regression controlling for age, sex, and cohort and using robust standard errors. St-ATT participants were not included in fat mass index analyses because of lack of data.

^2^ NPM, no prior malnutrition; PM, prior wasting malnutrition

**References**

1.	World Health Organization. Definition and diagnosis of diabetes mellitus and intermediate hyperglycaemia2006. Available from: <https://www.who.int/publications/i/item/definition-and-diagnosis-of-diabetes-mellitus-and-intermediate-hyperglycaemia>.

2.	World Health Organization. Use of Glycated Haemoglobin (HbA1c) in the Diagnosis of Diabetes Mellitus2011. Available from: <http://www.who.int/diabetes/publications/diagnosis_diabetes2011/en/>.

3.	Gibson RS. Principles of Nutritional Assessment. 2nd Edition ed. Oxford: Oxford University Press; 2005.

4.	van Abeelen AF, Elias SG, Bossuyt PM, Grobbee DE, van der Schouw YT, Roseboom TJ, et al. Famine exposure in the young and the risk of type 2 diabetes in adulthood. Diabetes. 2012;61(9):2255-2260.

5.	Li Y, He Y, Qi L, Jaddoe VW, Feskens EJ, Yang X, et al. Exposure to the Chinese famine in early life and the risk of hyperglycemia and type 2 diabetes in adulthood. Diabetes. 2010;59(10):2400-2406.

6.	Francis-Emmanuel PM, Thompson DS, Barnett AT, Osmond C, Byrne CD, Hanson MA, et al. Glucose metabolism in adult survivors of severe acute malnutrition. J Clin Endocrinol Metab. 2014;99(6):2233-2240.

7.	Ferdous F, Filteau S, Schwartz NB, Gumede-Moyo S, Cox SE. Association of postnatal severe acute malnutrition with pancreatic exocrine & endocrine function in children and adults: A systematic review. Br J Nutr. 2023;129:576-609.
